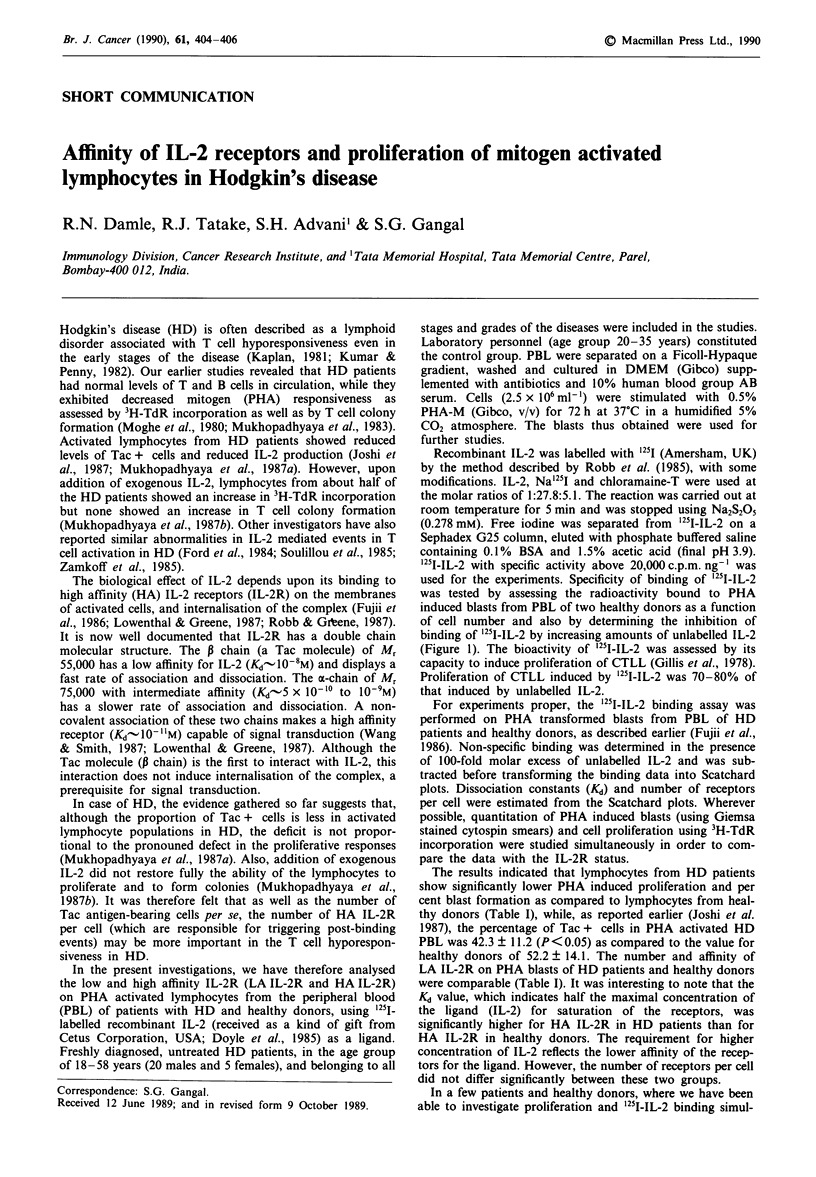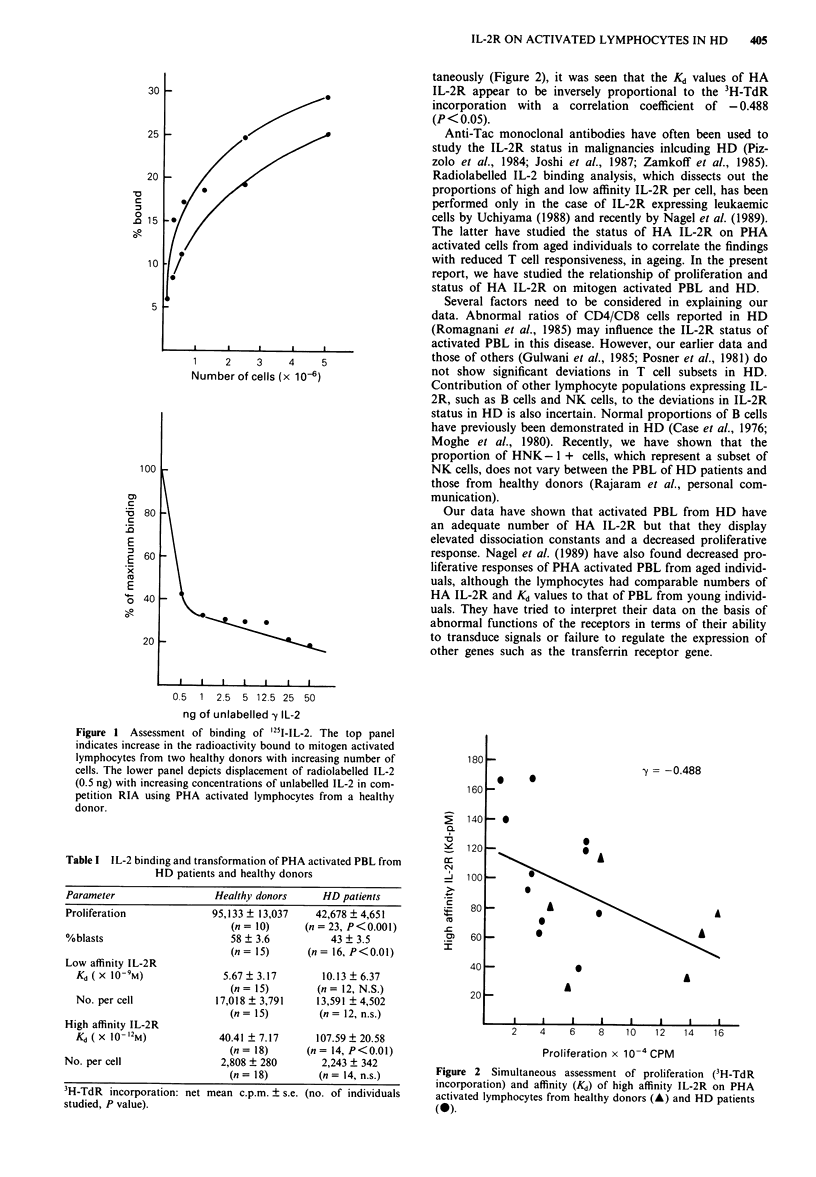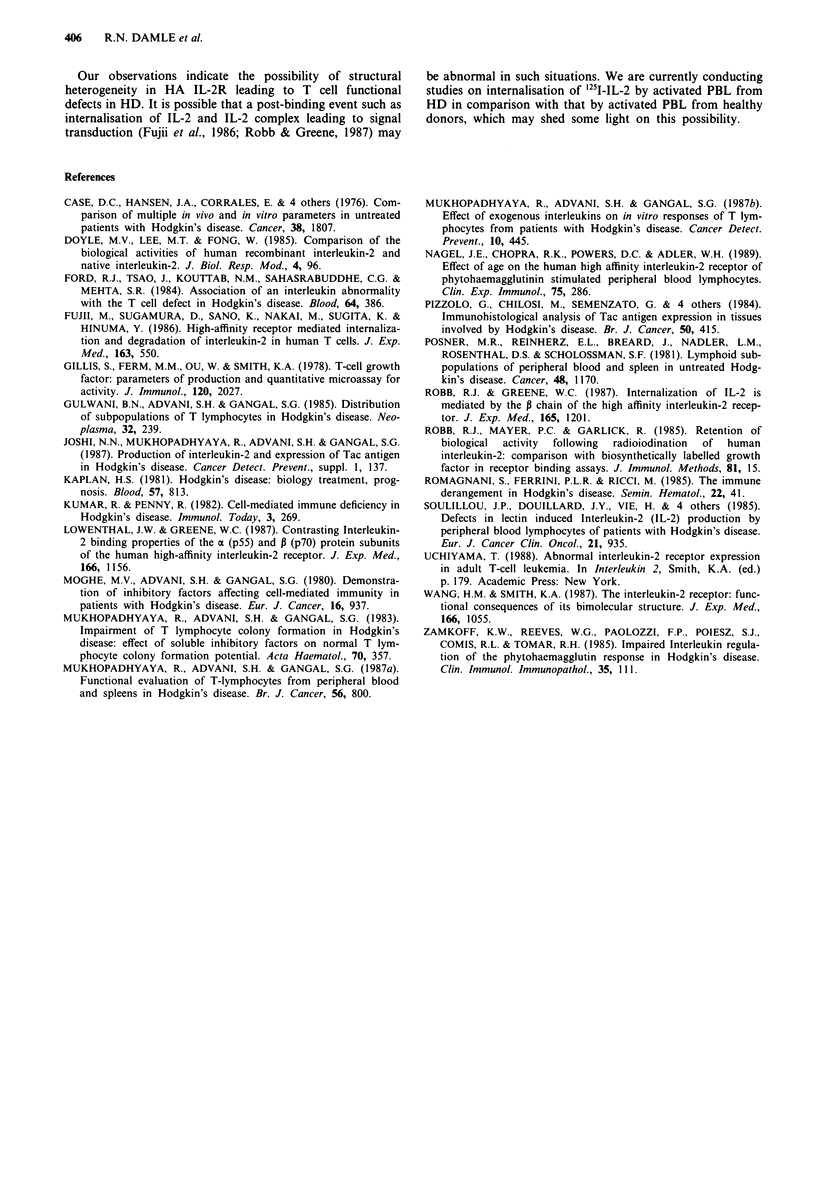# Affinity of IL-2 receptors and proliferation of mitogen activated lymphocytes in Hodgkin's disease.

**DOI:** 10.1038/bjc.1990.88

**Published:** 1990-03

**Authors:** R. N. Damle, R. J. Tatake, S. H. Advani, S. G. Gangal

**Affiliations:** Immunology Division, Cancer Research Institute, Parel, Bombay, India.


Br. J. Cancer (1990), 61, 404-406 © Macmillan Press Ltd., 1990

SHORT COMMUNICATION

### Affinity of IL-2 receptors and proliferation of mitogen activated lymphocytes in Hodgkin's disease

R.N. Damle, R.J. Tatake, S.H. Advani[1] & S.G. Gangal

*Immunology Division, Cancer Research Institute, and [1]Tata Memorial Hospital, Tata Memorial Centre, Parel, Bombay-400 012, India.*

Hodgkin's disease (HD) is often described as a lymphoid disorder associated with T cell hyporesponsiveness even in the early stages of the disease (Kaplan, 1981; Kumar & Penny, 1982). Our earlier studies revealed that HD patients had normal levels of T and B cells in circulation, while they exhibited decreased mitogen (PHA) responsiveness as assessed by $^3$H-TdR incorporation as well as by T cell colony formation (Moghe *et al.*, 1980; Mukhopadhyaya *et al.*, 1983). Activated lymphocytes from HD patients showed reduced levels of Tac + cells and reduced IL-2 production (Joshi *et al.*, 1987; Mukhopadhyaya *et al.*, 1987*a*). However, upon addition of exogenous IL-2, lymphocytes from about half of the HD patients showed an increase in $^3$H-TdR incorporation but none showed an increase in T cell colony formation (Mukhopadhyaya *et al.*, 1987*b*). Other investigators have also reported similar abnormalities in IL-2 mediated events in T cell activation in HD (Ford *et al.*, 1984; Soulillou *et al.*, 1985; Zamkoff *et al.*, 1985).

The biological effect of IL-2 depends upon its binding to high affinity (HA) IL-2 receptors (IL-2R) on the membranes of activated cells, and internalisation of the complex (Fujii *et al.*, 1986; Lowenthal & Greene, 1987; Robb & Greene, 1987). It is now well documented that IL-2R has a double chain molecular structure. The $\beta$ chain (a Tac molecule) of $M_r$ 55,000 has a low affinity for IL-2 ($K_d \sim 10^{-8}$M) and displays a fast rate of association and dissociation. The $\alpha$-chain of $M_r$ 75,000 with intermediate affinity ($K_d \sim 5 \times 10^{-10}$ to $10^{-9}$M) has a slower rate of association and dissociation. A non-covalent association of these two chains makes a high affinity receptor ($K_d \sim 10^{-11}$M) capable of signal transduction (Wang & Smith, 1987; Lowenthal & Greene, 1987). Although the Tac molecule ($\beta$ chain) is the first to interact with IL-2, this interaction does not induce internalisation of the complex, a prerequisite for signal transduction.

In case of HD, the evidence gathered so far suggests that, although the proportion of Tac + cells is less in activated lymphocyte populations in HD, the deficit is not proportional to the pronouned defect in the proliferative responses (Mukhopadhyaya *et al.*, 1987*a*). Also, addition of exogenous IL-2 did not restore fully the ability of the lymphocytes to proliferate and to form colonies (Mukhopadhyaya *et al.*, 1987*b*). It was therefore felt that as well as the number of Tac antigen-bearing cells *per se*, the number of HA IL-2R per cell (which are responsible for triggering post-binding events) may be more important in the T cell hyporesponsiveness in HD.

In the present investigations, we have therefore analysed the low and high affinity IL-2R (LA IL-2R and HA IL-2R) on PHA activated lymphocytes from the peripheral blood (PBL) of patients with HD and healthy donors, using $^{125}$I-labelled recombinant IL-2 (received as a kind of gift from Cetus Corporation, USA; Doyle *et al.*, 1985) as a ligand. Freshly diagnosed, untreated HD patients, in the age group of 18-58 years (20 males and 5 females), and belonging to all stages and grades of the diseases were included in the studies. Laboratory personnel (age group 20-35 years) constituted the control group. PBL were separated on a Ficoll-Hypaque gradient, washed and cultured in DMEM (Gibco) supplemented with antibiotics and 10% human blood group AB serum. Cells ($2.5 \times 10^6$ ml$^{-1}$) were stimulated with 0.5% PHA-M (Gibco, v/v) for 72 h at 37°C in a humidified 5% $CO_2$ atmosphere. The blasts thus obtained were used for further studies.

Recombinant IL-2 was labelled with $^{125}$I (Amersham, UK) by the method described by Robb *et al.* (1985), with some modifications. IL-2, Na$^{125}$I and chloramaine-T were used at the molar ratios of 1:27.8:5.1. The reaction was carried out at room temperature for 5 min and was stopped using $Na_2S_2O_5$ (0.278 mM). Free iodine was separated from $^{125}$I-IL-2 on a Sephadex G25 column, eluted with phosphate buffered saline containing 0.1% BSA and 1.5% acetic acid (final pH 3.9). $^{125}$I-IL-2 with specific activity above 20,000 c.p.m. ng$^{-1}$ was used for the experiments. Specificity of binding of $^{125}$I-IL-2 was tested by assessing the radioactivity bound to PHA induced blasts from PBL of two healthy donors as a function of cell number and also by determining the inhibition of binding of $^{125}$I-IL-2 by increasing amounts of unlabelled IL-2 (Figure 1). The bioactivity of $^{125}$I-IL-2 was assessed by its capacity to induce proliferation of CTLL (Gillis *et al.*, 1978). Proliferation of CTLL induced by $^{125}$I-IL-2 was 70-80% of that induced by unlabelled IL-2.

For experiments proper, the $^{125}$I-IL-2 binding assay was performed on PHA transformed blasts from PBL of HD patients and healthy donors, as described earlier (Fujii *et al.*, 1986). Non-specific binding was determined in the presence of 100-fold molar excess of unlabelled IL-2 and was subtracted before transforming the binding data into Scatchard plots. Dissociation constants ($K_d$) and number of receptors per cell were estimated from the Scatchard plots. Wherever possible, quantitation of PHA induced blasts (using Giemsa stained cytospin smears) and cell proliferation using $^3$H-TdR incorporation were studied simultaneously in order to compare the data with the IL-2R status.

The results indicated that lymphocytes from HD patients show significantly lower PHA induced proliferation and per cent blast formation as compared to lymphocytes from healthy donors (Table I), while, as reported earlier (Joshi *et al.* 1987), the percentage of Tac + cells in PHA activated HD PBL was 42.3 ± 11.2 ($P < 0.05$) as compared to the value for healthy donors of 52.2 ± 14.1. The number and affinity of LA IL-2R on PHA blasts of HD patients and healthy donors were comparable (Table I). It was interesting to note that the $K_d$ value, which indicates half the maximal concentration of the ligand (IL-2) for saturation of the receptors, was significantly higher for HA IL-2R in HD patients than for HA IL-2R in healthy donors. The requirement for higher concentration of IL-2 reflects the lower affinity of the receptors for the ligand. However, the number of receptors per cell did not differ significantly between these two groups.

In a few patients and healthy donors, where we have been able to investigate proliferation and $^{125}$I-IL-2 binding simul-

Correspondence: S.G. Gangal.
Received 12 June 1989; and in revised form 9 October 1989.

**Figure 1** Assessment of binding of $^{125}$I-IL-2. The top panel indicates increase in the radioactivity bound to mitogen activated lymphocytes from two healthy donors with increasing number of cells. The lower panel depicts displacement of radiolabelled IL-2 (0.5 ng) with increasing concentrations of unlabelled IL-2 in competition RIA using PHA activated lymphocytes from a healthy donor.

**Table I** IL-2 binding and transformation of PHA activated PBL from HD patients and healthy donors

| *Parameter* | *Healthy donors* | *HD patients* |
|---|---|---|
| Proliferation | 95,133 ± 13,037 ($n$ = 10) | 42,678 ± 4,651 ($n$ = 23, $P<0.001$) |
| %blasts | 58 ± 3.6 ($n$ = 15) | 43 ± 3.5 ($n$ = 16, $P<0.01$) |
| Low affinity IL-2R | | |
| $K_d$ ( × $10^{-9}$M) | 5.67 ± 3.17 ($n$ = 15) | 10.13 ± 6.37 ($n$ = 12, N.S.) |
| No. per cell | 17,018 ± 3,791 ($n$ = 15) | 13,591 ± 4,502 ($n$ = 12, n.s.) |
| High affinity IL-2R | | |
| $K_d$ ( × $10^{-12}$M) | 40.41 ± 7.17 ($n$ = 18) | 107.59 ± 20.58 ($n$ = 14, $P<0.01$) |
| No. per cell | 2,808 ± 280 ($n$ = 18) | 2,243 ± 342 ($n$ = 14, n.s.) |

$^3$H-TdR incorporation: net mean c.p.m. ± s.e. (no. of individuals studied, $P$ value).

taneously (Figure 2), it was seen that the $K_d$ values of HA IL-2R appear to be inversely proportional to the $^3$H-TdR incorporation with a correlation coefficient of −0.488 ($P<0.05$).

Anti-Tac monoclonal antibodies have often been used to study the IL-2R status in malignancies inlcuding HD (Pizzolo *et al.*, 1984; Joshi *et al.*, 1987; Zamkoff *et al.*, 1985). Radiolabelled IL-2 binding analysis, which dissects out the proportions of high and low affinity IL-2R per cell, has been performed only in the case of IL-2R expressing leukaemic cells by Uchiyama (1988) and recently by Nagel *et al.* (1989). The latter have studied the status of HA IL-2R on PHA activated cells from aged individuals to correlate the findings with reduced T cell responsiveness, in ageing. In the present report, we have studied the relationship of proliferation and status of HA IL-2R on mitogen activated PBL and HD.

Several factors need to be considered in explaining our data. Abnormal ratios of CD4/CD8 cells reported in HD (Romagnani *et al.*, 1985) may influence the IL-2R status of activated PBL in this disease. However, our earlier data and those of others (Gulwani *et al.*, 1985; Posner *et al.*, 1981) do not show significant deviations in T cell subsets in HD. Contribution of other lymphocyte populations expressing IL-2R, such as B cells and NK cells, to the deviations in IL-2R status in HD is also incertain. Normal proportions of B cells have previously been demonstrated in HD (Case *et al.*, 1976; Moghe *et al.*, 1980). Recently, we have shown that the proportion of HNK−1+ cells, which represent a subset of NK cells, does not vary between the PBL of HD patients and those from healthy donors (Rajaram *et al.*, personal communication).

Our data have shown that activated PBL from HD have an adequate number of HA IL-2R but that they display elevated dissociation constants and a decreased proliferative response. Nagel *et al.* (1989) have also found decreased proliferative responses of PHA activated PBL from aged individuals, although the lymphocytes had comparable numbers of HA IL-2R and $K_d$ values to that of PBL from young individuals. They have tried to interpret their data on the basis of abnormal functions of the receptors in terms of their ability to transduce signals or failure to regulate the expression of other genes such as the transferrin receptor gene.

**Figure 2** Simultaneous assessment of proliferation ($^3$H-TdR incorporation) and affinity ($K_d$) of high affinity IL-2R on PHA activated lymphocytes from healthy donors (▲) and HD patients (●).

Our observations indicate the possibility of structural heterogeneity in HA IL-2R leading to T cell functional defects in HD. It is possible that a post-binding event such as internalisation of IL-2 and IL-2 complex leading to signal transduction (Fujii et al., 1986; Robb & Greene, 1987) may be abnormal in such situations. We are currently conducting studies on internalisation of $^{125}$I-IL-2 by activated PBL from HD in comparison with that by activated PBL from healthy donors, which may shed some light on this possibility.

### References

CASE, D.C., HANSEN, J.A., CORRALES, E. & 4 others (1976). Comparison of multiple *in vivo* and *in vitro* parameters in untreated patients with Hodgkin's disease. *Cancer*, **38**, 1807.

DOYLE, M.V., LEE, M.T. & FONG, W. (1985). Comparison of the biological activities of human recombinant interleukin-2 and native interleukin-2. *J. Biol. Resp. Mod.*, **4**, 96.

FORD, R.J., TSAO, J., KOUTTAB, N.M., SAHASRABUDDHE, C.G. & MEHTA, S.R. (1984). Association of an interleukin abnormality with the T cell defect in Hodgkin's disease. *Blood*, **64**, 386.

FUJII, M., SUGAMURA, D., SANO, K., NAKAI, M., SUGITA, K. & HINUMA, Y. (1986). High-affinity receptor mediated internalization and degradation of interleukin-2 in human T cells. *J. Exp. Med.*, **163**, 550.

GILLIS, S., FERM, M.M., OU, W. & SMITH, K.A. (1978). T-cell growth factor: parameters of production and quantitative microassay for activity. *J. Immunol.*, **120**, 2027.

GULWANI, B.N., ADVANI, S.H. & GANGAL, S.G. (1985). Distribution of subpopulations of T lymphocytes in Hodgkin's disease. *Neoplasma*, **32**, 239.

JOSHI, N.N., MUKHOPADHYAYA, R., ADVANI, S.H. & GANGAL, S.G. (1987). Production of interleukin-2 and expression of Tac antigen in Hodgkin's disease. *Cancer Detect. Prevent.*, suppl. 1, 137.

KAPLAN, H.S. (1981). Hodgkin's disease: biology treatment, prognosis. *Blood*, **57**, 813.

KUMAR, R. & PENNY, R. (1982). Cell-mediated immune deficiency in Hodgkin's disease. *Immunol. Today*, **3**, 269.

LOWENTHAL, J.W. & GREENE, W.C. (1987). Contrasting Interleukin-2 binding properties of the α (p55) and β (p70) protein subunits of the human high-affinity interleukin-2 receptor. *J. Exp. Med.*, **166**, 1156.

MOGHE, M.V., ADVANI, S.H. & GANGAL, S.G. (1980). Demonstration of inhibitory factors affecting cell-mediated immunity in patients with Hodgkin's disease. *Eur. J. Cancer*, **16**, 937.

MUKHOPADHYAYA, R., ADVANI, S.H. & GANGAL, S.G. (1983). Impairment of T lymphocyte colony formation in Hodgkin's disease: effect of soluble inhibitory factors on normal T lymphocyte colony formation potential. *Acta Haematol.*, **70**, 357.

MUKHOPADHYAYA, R., ADVANI, S.H. & GANGAL, S.G. (1987*a*). Functional evaluation of T-lymphocytes from peripheral blood and spleens in Hodgkin's disease. *Br. J. Cancer*, **56**, 800.

MUKHOPADHYAYA, R., ADVANI, S.H. & GANGAL, S.G. (1987*b*). Effect of exogenous interleukins on *in vitro* responses of T lymphocytes from patients with Hodgkin's disease. *Cancer Detect. Prevent.*, **10**, 445.

NAGEL, J.E., CHOPRA, R.K., POWERS, D.C. & ADLER, W.H. (1989). Effect of age on the human high affinity interleukin-2 receptor of phytohaemagglutinin stimulated peripheral blood lymphocytes. *Clin. Exp. Immunol.*, **75**, 286.

PIZZOLO, G., CHILOSI, M., SEMENZATO, G. & 4 others (1984). Immunohistological analysis of Tac antigen expression in tissues involved by Hodgkin's disease. *Br. J. Cancer*, **50**, 415.

POSNER, M.R., REINHERZ, E.L., BREARD, J., NADLER, L.M., ROSENTHAL, D.S. & SCHOLOSSMAN, S.F. (1981). Lymphoid subpopulations of peripheral blood and spleen in untreated Hodgkin's disease. *Cancer*, **48**, 1170.

ROBB, R.J. & GREENE, W.C. (1987). Internalization of IL-2 is mediated by the β chain of the high affinity interleukin-2 receptor. *J. Exp. Med.*, **165**, 1201.

ROBB, R.J., MAYER, P.C. & GARLICK, R. (1985). Retention of biological activity following radioiodination of human interleukin-2: comparison with biosynthetically labelled growth factor in receptor binding assays. *J. Immunol. Methods*, **81**, 15.

ROMAGNANI, S., FERRINI, P.L.R. & RICCI, M. (1985). The immune derangement in Hodgkin's disease. *Semin. Hematol.*, **22**, 41.

SOULILLOU, J.P., DOUILLARD, J.Y., VIE, H. & 4 others (1985). Defects in lectin induced Interleukin-2 (IL-2) production by peripheral blood lymphocytes of patients with Hodgkin's disease. *Eur. J. Cancer Clin. Oncol.*, **21**, 935.

UCHIYAMA, T. (1988). Abnormal interleukin-2 receptor expression in adult T-cell leukemia. In *Interleukin 2*, Smith, K.A. (ed.) p. 179. Academic Press: New York.

WANG, H.M. & SMITH, K.A. (1987). The interleukin-2 receptor: functional consequences of its bimolecular structure. *J. Exp. Med.*, **166**, 1055.

ZAMKOFF, K.W., REEVES, W.G., PAOLOZZI, F.P., POIESZ, S.J., COMIS, R.L. & TOMAR, R.H. (1985). Impaired Interleukin regulation of the phytohaemagglutin response in Hodgkin's disease. *Clin. Immunol. Immunopathol.*, **35**, 111.